# Prognostic value of the ferroptosis-related gene *SLC2A3* in gastric cancer and related immune mechanisms

**DOI:** 10.3389/fgene.2022.919313

**Published:** 2022-07-25

**Authors:** Liubing Lin, Renye Que, Jian Wang, Yi Zhu, Xiaolin Liu, Rongzhong Xu

**Affiliations:** ^1^ Department of Gastroenterology, Shanghai Municipal Hospital of Traditional Chinese Medicine, Shanghai University of Traditional Chinese Medicine, Shanghai, China; ^2^ Department of Gastroenterology, Shanghai TCM Integrated Hospital, Shanghai University of Traditional Chinese Medicine, Shanghai, China; ^3^ Department of Internal Medicine I, Shanghai Jiading District Hospital of Traditional Chinese Medicine, Shanghai, China; ^4^ Department of Oncology, Shanghai Municipal Hospital of Traditional Chinese Medicine, Shanghai University of Traditional Chinese Medicine, Shanghai, China

**Keywords:** SLC2A3, ferroptosis, prognosis, immune, gastric carcinoma

## Abstract

SLC2A3 is a ferroptosis marker engaged in transmembrane glucose transport. However, the effect of SLC2A3 on the prognosis of patients with cancer remains unclear. This study aimed to explore the prognostic implications of SLC2A3 and its underlying immune mechanisms in gastric cancer. The mRNA expression profiles and corresponding clinical data of patients with gastric cancer were downloaded from The Cancer Genome Atlas and Gene Expression Omnibus databases. Differentially expressed genes related to SLC2A3 were identified using the R package “limma.” Gene ontology and Kyoto Encyclopedia of Genes and Genomes enrichment analyses, gene set enrichment analysis, and gene set variation analysis were used to explore the underlying mechanisms. The protein–protein and miRNA interaction networks were analyzed using Cytoscape software. Immune cell infiltration was assessed using single-sample gene set enrichment analysis. Univariate and multivariate Cox regression analyses revealed the relationship between SLC2A3 expression and prognosis. SLC2A3 was found to be highly expressed in tumor tissues and was associated with an unfavorable prognosis in patients with gastric cancer. Functional enrichment analysis showed that SLC2A3 is related to cytokine–cytokine receptor interaction, epithelial–mesenchymal transition, T cell receptor signaling pathway, B cell receptor signaling pathway, and immune checkpoints. SLC2A3 is also involved in immune response regulation and is regulated by multiple miRNAs, including miR-195-5p, miR-106a-5p, miR-424-5p, and miR-16-5p. Univariate and multivariate Cox regression analyses indicated that SLC2A3 can be used as an independent prognostic factor for predicting the outcome in patients with gastric cancer. SLC2A3 and related miRNAs are potential prognostic biomarkers and therapeutic targets.

## Introduction

Gastric cancer is a gastrointestinal malignancy that accounts for 17.2% of all malignant tumors, and it is associated with the second-highest fatality rate among all common tumors worldwide (Thrift et al., 2020). Owing to the continuous improvement in the level of medical diagnosis in recent years, the diagnosis rate of gastric cancer has also increased (Sumiyama, 2017). Although advances in medicine have led to development of treatments for gastric cancer, the overall clinical outcomes of gastric cancer are still poor, with rapid development and poor treatment efficacy (Smyth et al., 2020). In recent years, with the continuous development and growth of molecular biology and genomics, screening for novel prognostic markers has gradually become a new strategy for predicting the prognosis of patients and also contributes to the treatment of patients with cancer.

SLC2A3 encodes solute carrier family 2-member 3 (also known as GLUT3, a ferroptosis marker), which mainly engages in transmembrane transport (e.g., for glucose transport) (Reckzeh and Waldmann, 2020). SLC2A3 functions as a tumor promoter and accelerates aerobic glycolysis in gastric cancer cells, and it potentially contributes to the M2 subtype transition of infiltrating macrophages in the microenvironment of gastric cancer. Thus, SLC2A3 may be useful as a biomarker to determine the prognosis and immune infiltration (Yao et al., 2020). Inhibition of SLC2A3 by miR-129-5p suppresses glucose consumption in gastric cancer cells by regulating the PI3K-Akt and MAPK signaling pathways, suggesting that SLC2A3 is a potential therapeutic target for the treatment of gastric cancer (Chen et al., 2018). These findings indicate that SLC2A3 may have a potential role in the prognosis of gastric cancer and regulation of tumor-infiltrating immune cells. However, whether SLC2A3 affects the prognosis of gastric cancer has not yet been systematically or elaborately elucidated.

This study aimed to determine the prognostic value of SLC2A3 expression in gastric cancer. We found that SLC2A3, as a ferroptosis marker, was highly expressed in gastric cancer tissues and was associated with poor prognosis in patients with gastric cancer, and it contributed through immune-related pathways.

## Materials and methods

### Data acquisition and preprocessing

The transcriptome data of gastric cancer in counts and FPKM formats were downloaded from The Cancer Genome Atlas-Stomach Adenocarcinoma (TCGA-STAD, https://portal.gdc.cancer.gov) database using the R package “TCGAbiolinks” (version 3.6.5, http://r-project.org/) (Mounir et al., 2019), and the data in FPKM format were converted to the TPM format. Clinical information of 375 patients with gastric cancer was also acquired from the TCGA database.

Reliable datasets GSE66229 (Oh et al., 2018) and GSE12266 (Granchi et al., 2010) were downloaded from the Gene Expression Omnibus (GEO; https://www.ncbi.nlm.nih.gov/geo/) database using the R package “GEOquery” (version 3.6.5, http://r-project.org/) (Davis and Meltzer, 2007). The samples in the datasets were all from *Homo sapiens*, and the platform was based on the GPL570 [HG-U133_Plus_2] Affymetrix Human Genome U133 Plus 2.0 Array. The GSE66229 dataset contains two GEO datasets: GSE62254 (Cristescu et al., 2015) and GSE66222. The GSE62254 dataset included 300 gastric cancer samples, and the GSE66222 dataset included 100 normal control samples. The GSE12266 dataset contained a total of 200 gastric cancer samples, of which eight were eliminated due to quality issues. Therefore, in the GEO database, 592 samples were included in this study and divided into the control (100 samples) and tumor groups (492 samples). The abovementioned samples were subjected to background correction and data normalization to obtain the gene expression matrices of the two datasets. The R package “sva” was used to remove the batch effects and obtain the combined gene expression matrix.

### Analysis of SLC2A3 expression in gastric cancer

In the TCGA and GEO datasets, SLC2A3 expression in gastric cancer and adjacent normal tissues was analyzed, and the results were visualized using the R package “ggplot2” (https://ggplot2.tidyverse.org). The R package “survminer” (https://CRAN.R-project.org/package=survminer) was used to select the optimal cutoff value for grouping according to the expression level of SLC2A3, and the R package “survival” (https://CRAN.R-project.org/package=survival) was used to draw a survival curve.

The expression data for gastric cancer were downloaded from the Genotype-Tissue Expression database (https://gtexportal.org/home/) to analyze the expression of SLC2A3 in various organs. In the TCGA database, pan-cancer FPKM data were downloaded and converted into the TPM format to analyze the differences in SLC2A3 expression in different tumors and adjacent normal tissues.

### Screening and functional analysis of differentially expressed genes

In both TCGA and GEO datasets, the tumor samples were grouped according to the median of SLC2A3 expression and the DEGs of the gene expression matrix were screened using the R package “limma” (Ritchie et al., 2015), with the criteria of *p* < 0.05 and |logFC|>0.5. When the value of |logFC| was 1, 1.5, or 2, the GEO dataset would obtain very few DEGs because the threshold was too high. In order to keep the threshold consistent, we took |logFC| > 0.5 both in the TCGA and GEO datasets (Huang et al., 2021; Bruzas et al., 2022). The volcano map and heat map of DEGs were drawn using the “ggplot2” R package to visualize the differential expression of DEGs.

The “clusterProfiler” R package (Yu et al., 2012) was used to perform the Gene Ontology (GO) and Kyoto Encyclopedia of Genes and Genomes (KEGG) enrichment analysis on DEGs. Similarly, the “clusterProfiler” R package was also used to conduct the gene set enrichment analysis (GSEA) analysis on DEGs. The “c2. cp.kegg.v7.0. entrez.gmt” gene set was selected as the reference. The false discovery rate (FDR) < 0.25 and *p* < 0.05 were considered to be significantly enriched.

### Gene set variation analysis

In the TCGA dataset, the R package “GSVA” (Hänzelmann et al., 2013) was used to perform GSVA on the expression matrix, and the “c2. cp.kegg.v7.0. Entrez. gmt” gene set was used as the reference. The tumor samples were grouped according to the median of SLC2A3 expression, and the differential pathways between the high- and low-expression groups were analyzed using the R package “limma.” The top 10 pathways with significant differences were selected and visualized as a heat map drawn using the R package “pheatmap.” Eighteen genes in the common biological function pathway were used as reference gene sets for GSVA. We also determined differences in this pathway between the high- and low-expression groups, and the results were visualized using the R package “ggplot2”.

### Protein–protein interaction and miRNA interaction networks

Protein interaction network analysis on SLC2A3 was performed using the STRING database (https://string-db.org/) (Szklarczyk et al., 2017) and visualized using Cytoscape software. The Cytohubba plugin was used to calculate the most important genes and to show the protein interaction network.

The R package “multiMiR” (Ru et al., 2014) was used to predict miRNAs that interacted with SLC2A3, and the intersection of these miRNAs with those in the miRTarBase database was taken. The obtained prediction results were visualized using Cytoscape software. The miRNAs whose verification was “Luciferase Reporter Gene Experiment” were selected and used to analyze their expression in tumor and adjacent normal tissues.

### Immune cell infiltration analysis

Single-sample gene set enrichment analysis (ssGSEA) can calculate the rank value of each gene according to the gene expression profile and then statistical analysis was performed (Bindea et al., 2013). We analyzed the gene expression matrix and cell markers to obtain an immune cell infiltration matrix. The fraction of 24 immune cells in the high- and low-expression groups was shown by R software.

Tumor microenvironment cells and the extent to which infiltrating immune and stromal cells in the tumor contribute significantly to prognosis are analyzed. In the tumor microenvironment, immune and stromal cells are the two main types of non-tumor components and have been proposed to be valuable for the diagnosis and prognostic evaluation of tumors (Zhang et al., 2020). The immune and stromal scores calculated based on the ESTIMATE algorithm can facilitate the quantification of immune and stromal components in tumors. In this algorithm, immune and stromal scores were calculated by analyzing specific gene expression signatures of immune and stromal cells to predict the infiltration of non-tumor cells. In addition, we calculated the StromalScore, ImmuneScore, and ESTIMATEScore of each sample using the R package “ESTIMATE” (Yoshihara et al., 2013) and the correlation between the expression level of SLC2A3 and each score was shown as a scatter plot. Differences in scores between the high- and low-expression groups were also analyzed.

### Clinical model construction

Univariate and multivariate Cox regression analyses were conducted using the R package “survival” based on SLC2A3 expression and some clinical variables (T stage, N stage, M stage, pathologic stage, gender; age, histologic grade, and residual tumor), and the results are shown as forest diagrams. In addition, in some subgroups, we analyzed the effect of SLC2A3 expression on the survival of patients with gastric cancer.

A clinical prognostic model was analyzed and constructed using the R packages “rms” (https://CRAN.R-project.org/package=rms) and “survival,” and a nomogram was constructed. In addition, we performed calibration analysis to evaluate the predictive ability of the model.

### Statistical analysis

R software (version 4.1.0, https://www.r-project.org/) was used for statistical analyses. The independent *t*-test was used for comparison of normally distributed variables, and the Mann–Whitney *U* test was used for comparison of non-normally distributed variables. All statistical tests were two-sided, and statistical significance was set at *p* < 0.05.

## Results

### SLC2A3 is highly expressed and is a poor prognostic factor in gastric cancer

**FIGURE 1:**
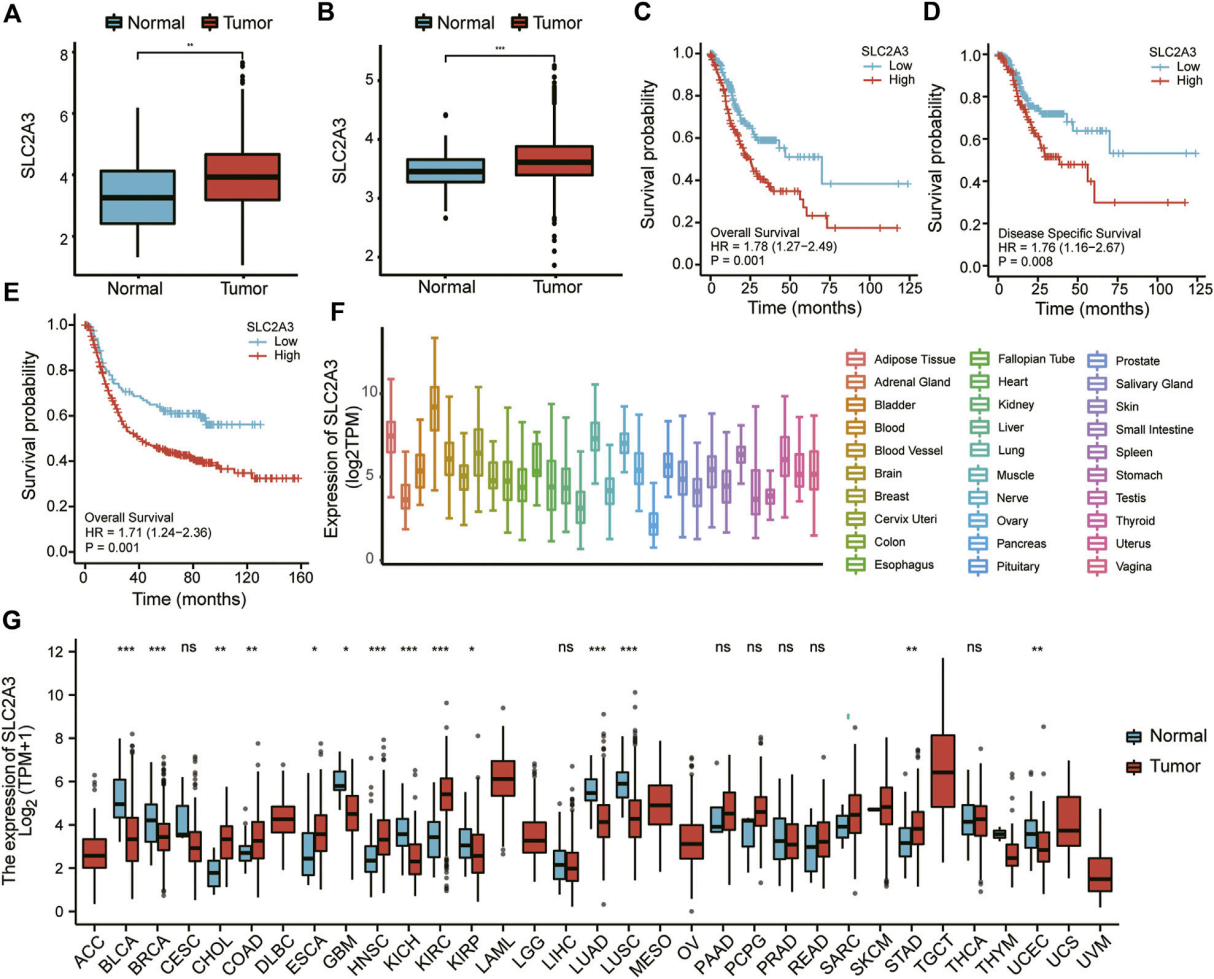
SLC2A3 was highly expressed and was a prognostic factor for poor outcomes in gastric cancer. Expression of SLC2A3 in TCGA **(A)** and GEO **(B)** databases. The relationship of SLC2A3 expression with OS **(C)** or DSS **(D)** in the TCGA database and OS **(E)** in the GEO database. Expression of SLC2A3 in various organs **(F)** and pan-carcinomas **(G)** in the TCGA database.

First, the expression of SLC2A3 was analyzed in tumor and adjacent normal tissues in the TCGA and combined GEO datasets. As shown in Figures 1A,B, compared with the adjacent normal tissues, SLC2A3 was highly expressed in tumor tissues in both TCGA (*p* < 0.001) and GEO datasets (*p* < 0.0001). In the TCGA dataset, survival analysis indicated that a high expression level of SLC2A3 was significantly associated with poor prognosis in patients with gastric cancer, both in overall survival (OS; *p* = 0.001; Figure 1C) and disease-specific survival (DSS; *p* = 0.008; Figure 1D). In the GEO dataset, we obtained similar results; the high expression level of SLC2A3 was significantly associated with poor prognosis in patients with OS (*p* = 0.001; Figure 1E). These results suggest that SLC2A3 is highly expressed in tumor tissues and is related to poor prognosis in patients with gastric cancer.

We also analyzed the expression of SLC2A3 in different organs and pan-carcinomas using the TCGA database. The results showed that SLC2A3 had the highest expression in blood (Figure 1F). Compared with adjacent normal tissues, SLC2A3 was highly expressed in gastric cancer, colon cancer, and other tumors (*p* < 0.05; Figure 1G). These results further suggest that SLC2A3 is highly expressed in gastric cancer tissues.

Then, according to the median value of SLC2A3 expression, 375 patients with complete clinical data in the TCGA database were divided into high- and low-expression groups. The T stages of patients were significantly different between the two groups (*p* = 0.002; Table 1), suggesting that high SLC2A3 expression is associated with the T stage in patients with gastric cancer.

**TABLE 1:** Clinical traits between SLC2A3 high- and low-expression groups.

Characteristic	Levels	Low expression of SLC2A3	High expression of SLC2A3	p
N		187	188	
Age, n (%)	≤65	88 (23.7%)	76 (20.5%)	0.312
	>65	99 (26.7%)	108 (29.1%)	
Gender, n (%)	Female	66 (17.6%)	68 (18.1%)	0.945
	Male	121 (32.3%)	120 (32%)	
T stage, n (%)	T1	16 (4.4%)	3 (0.8%)	0.002
	T2	40 (10.9%)	40 (10.9%)	
	T3	92 (25.1%)	76 (20.7%)	
	T4	39 (10.6%)	61 (16.6%)	
N stage, n (%)	N0	58 (16.2%)	53 (14.8%)	0.577
	N1	50 (14%)	47 (13.2%)	
	N2	40 (11.2%)	35 (9.8%)	
	N3	32 (9%)	42 (11.8%)	
M stage, n (%)	M0	167 (47%)	163 (45.9%)	0.965
	M1	12 (3.4%)	13 (3.7%)	
Pathologic stage, n (%)	Stage I	32 (9.1%)	21 (6%)	0.256
	Stage II	59 (16.8%)	52 (14.8%)	
	Stage III	71 (20.2%)	79 (22.4%)	
	Stage IV	16 (4.5%)	22 (6.2%)	
Residual tumor, n (%)	R0	152 (46.2%)	146 (44.4%)	0.616
	R1	6 (1.8%)	9 (2.7%)	
	R2	7 (2.1%)	9 (2.7%)	
Histologic grade, n (%)	G1	6 (1.6%)	4 (1.1%)	0.082
	G2	77 (21%)	60 (16.4%)	
	G3	98 (26.8%)	121 (33.1%)	

### Screening of DEGs

**FIGURE 2:**
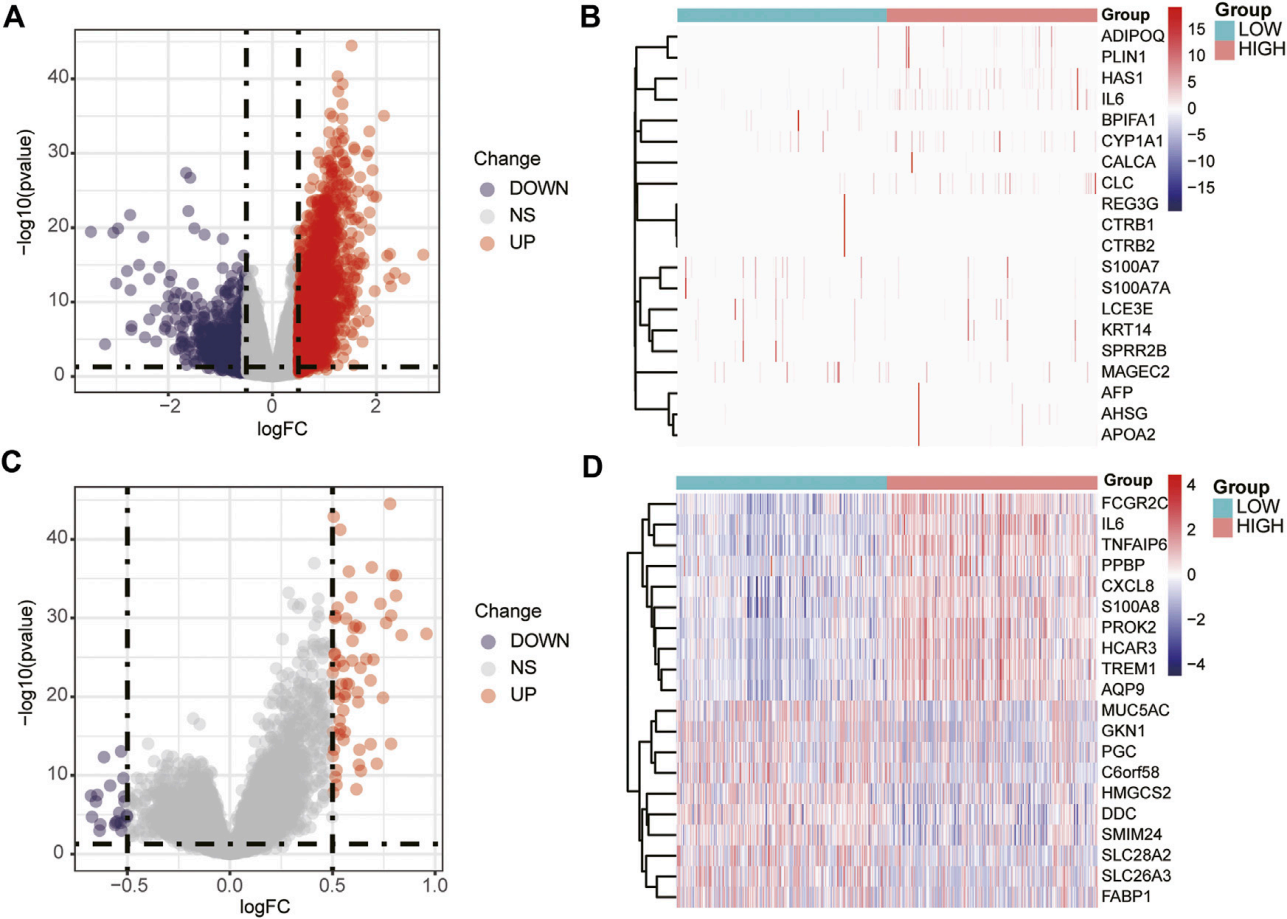
Volcano plot and heat map of DEGs. Volcano plot **(A)** and heat map **(B)** of DEGs screened in the TCGA database. Volcano plot **(C)** and heat map **(D)** of the DEGs screened in the GEO database.

A total of 3,135 DEGs between the high- and low-expression groups were obtained from the TCGA database and are shown as a volcano plot (Figure 2A). The top 20 genes with significant differences are shown in a heatmap (Figure 2B). A similar screening method was applied to the GEO database, and the volcano plot and heat map are shown in Figures 2C,D, respectively.

### Functional enrichment analysis of DEGs by Gene ontology and Kyoto Encyclopedia of Genes and Genomes

**FIGURE 3:**
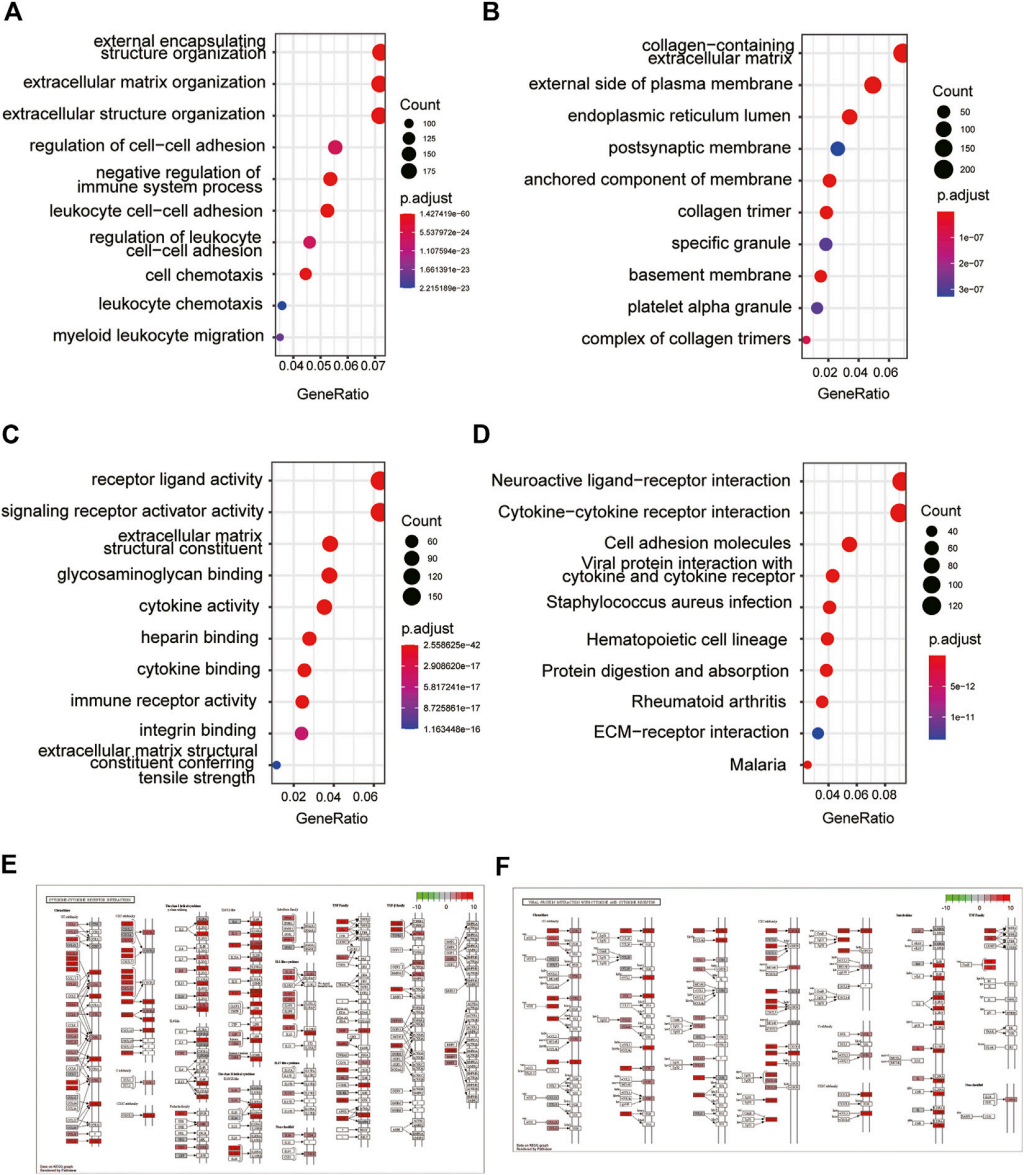
GO and KEGG enrichment analysis of DEGs. GO enrichment analysis of DEGs in GO_BP **(A)**, GO_CC **(B)**, and GO_MF **(C)**. KEGG enrichment analysis of DEGs **(D)**. Exhibition of cytokine–cytokine receptor interaction **(E)** and viral protein interaction with cytokine and cytokine receptors **(F)**.

To explore the biological functions of SLC2A3, we further performed GO and KEGG enrichment analysis on DEGs obtained from the TCGA database. GO_BP analysis results showed that DEGs were mainly related to external encapsulating structure organization, extracellular matrix organization, and extracellular structure organization (Table 2 and Figure 3A). GO_CC analysis results showed that DEGs were mainly associated with the collagen-containing extracellular matrix, external side of the plasma membrane, and the endoplasmic reticulum lumen. (Table 2; Figure 3B). Meanwhile, GO_MF analysis results showed that DEGs were mainly related to receptor-ligand activity, signaling receptor activator activity, and extracellular matrix structural constituents. (Table 2; Figure 3C).

**TABLE 2:** GO enrichment of DEGs.

Ontology	ID	Description	P value	Q value
BP	GO:0,045,229	External encapsulating structure organization	2.44E-64	1.08E-60
BP	GO:0,030,198	Extracellular matrix organization	2.95E-64	1.08E-60
BP	GO:0,043,062	Extracellular structure organization	5.12E-64	1.25E-60
BP	GO:0,007,159	Leukocyte cell-cell adhesion	3.16E-32	5.81E-29
BP	GO:0,002,683	Negative regulation of immune system process	1.12E-28	1.65E-25
CC	GO:0,062,023	Collagen-containing extracellular matrix	3.25E-59	2.55E-56
CC	GO:0,009,897	External side of plasma membrane	6.10E-26	2.40E-23
CC	GO:0,005,581	Collagen trimer	1.49E-24	3.89E-22
CC	GO:0,005,788	Endoplasmic reticulum lumen	5.38E-15	1.06E-12
CC	GO:0,005,604	Basement membrane	2.52E-13	3.96E-11
MF	GO:0,005,201	Extracellular matrix structural constituent	1.25E-45	2.24E-42
MF	GO:0,048,018	Receptor ligand activity	3.27E-31	2.93E-28
MF	GO:0,030,546	Signaling receptor activator activity	1.88E-30	1.12E-27
MF	GO:0,005,539	Glycosaminoglycan binding	3.10E-29	1.39E-26
MF	GO:0,019,955	Cytokine binding	9.24E-24	3.30E-21

The results of KEGG analysis suggested that the pathways enriched by DEGs mainly included neuroactive ligand–receptor interaction, cytokine–cytokine receptor interaction, and cell adhesion molecules (Table 3; Figure 3D). The two pathways with the best enrichment results were cytokine–cytokine receptor interaction (Figure 3E) and viral protein interaction with cytokines and cytokine receptors (Figure 3F). Among these, CCL2, CCL19, and other genes play an important role in the signal transduction process of the two pathways.

**TABLE 3:** KEGG enrichment of DEGs.

ID	Description	P value	Q value	Count
hsa04060	Cytokine–cytokine receptor interaction	1.03E-24	2.34E-22	122
hsa04061	Viral protein interaction with cytokine and cytokine receptor	5.48E-21	4.57E-19	58
hsa04514	Cell adhesion molecules	6.03E-21	4.57E-19	74
hsa05150	*Staphylococcus aureus* infection	1.28E-19	7.29E-18	55
hsa04080	Neuroactive ligand–receptor interaction	2.18E-19	9.91E-18	124
hsa04640	Hematopoietic cell lineage	3.52E-17	1.33E-15	53
hsa05144	Malaria	7.79E-16	2.53E-14	34
hsa04974	Protein digestion and absorption	1.83E-15	5.21E-14	52
hsa05323	Rheumatoid arthritis	7.41E-15	1.87E-13	48
hsa04512	ECM–receptor interaction	4.29E-13	9.75E-12	44

### Functional enrichment analysis of DEGs by gene set enrichment analysis

**FIGURE 4:**
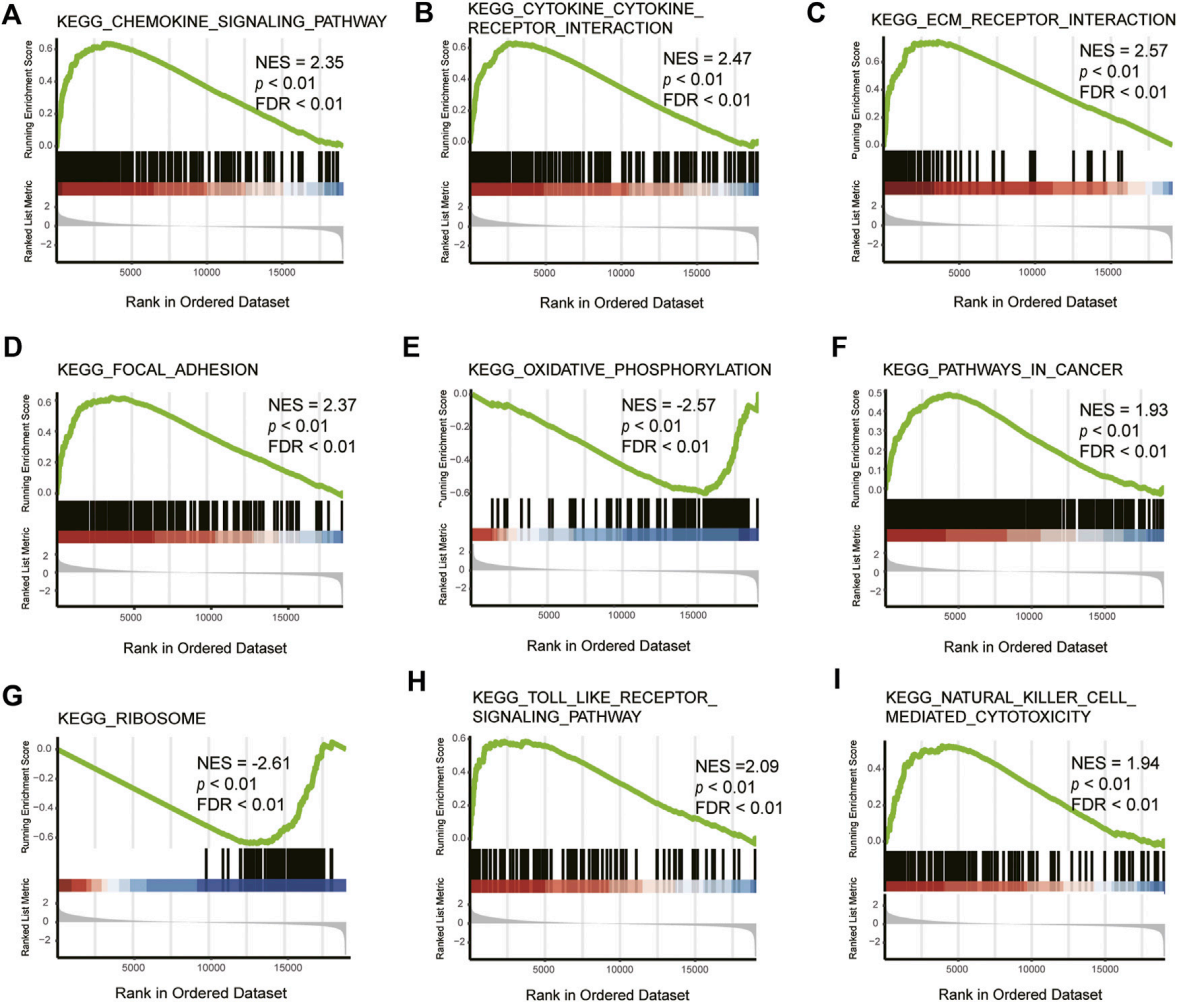
GSEA of DEGs. NES: normalized enrichment score, FDR: false discovery rate. KEGG_CHEMOKINE_SIGNALING_PATHWAY **(A)**, KEGG_CYTOKINE_CYTOKINE_RECEPTOR_INTERACTION **(B)**, KEGG_ECM_RECEPTOR_INTERACTION **(C)**, KEGG_FOCAL_ADHESION **(D)**, KEGG_OXIDATIVE_PHOSPHORYLATION **(E)**, KEGG_PATHWAYS_IN_CANCER **(F)**, KEGG_RIBOSOME **(G)**, KEGG_TOLL_LIKE_RECEPTOR_SIGNALING_PATHWAY **(H)**, KEGG_NATURAL_KILLER_CELL_MEDIATED_CYTOTOXICITY **(I)**.

To further explore the function of DEGs, GSEA analysis was conducted and it was found that the pathways mainly involved were the chemokine signaling pathway, cytokine-cytokine receptor interaction, ECM receptor interaction, focal adhesion, oxidative phosphorylation, pathways in cancer, Toll-like receptor signaling pathway, and natural killer cell-mediated cytotoxicity pathways, of which ECM receptor interaction, cytokine–cytokine receptor interaction, and Toll-like receptor signaling pathway pathways were closely related to ferroptosis (Table 4, Figure 4).

**TABLE 4:** Pathway enrichment in GSEA.

ID	SetSize	NES	*P* value	*Q* values
KEGG_CELL_ADHESION_MOLECULES_CAMS	130	2.347,126,682	1.00E-10	8.42E-10
KEGG_CHEMOKINE_SIGNALING_PATHWAY	187	2.423,098,421	1.00E-10	8.42E-10
KEGG_CYTOKINE_CYTOKINE_RECEPTOR_INTERACTION	262	2.474,431,198	1.00E-10	8.42E-10
KEGG_ECM_RECEPTOR_INTERACTION	83	2.575,153,081	1.00E-10	8.42E-10
KEGG_FOCAL_ADHESION	199	2.370,966,486	1.00E-10	8.42E-10
KEGG_HEMATOPOIETIC_CELL_LINEAGE	84	2.527,143,211	1.00E-10	8.42E-10
KEGG_LEISHMANIA_INFECTION	69	2.388,049,815	1.00E-10	8.42E-10
KEGG_OXIDATIVE_PHOSPHORYLATION	116	−2.575,690,856	1.00E-10	8.42E-10
KEGG_PARKINSONS_DISEASE	113	−2.419,185,523	1.00E-10	8.42E-10
KEGG_PATHWAYS_IN_CANCER	325	1.937,317,481	1.00E-10	8.42E-10

### Pathway enrichment analysis of DEGs by GSVA

**FIGURE 5:**
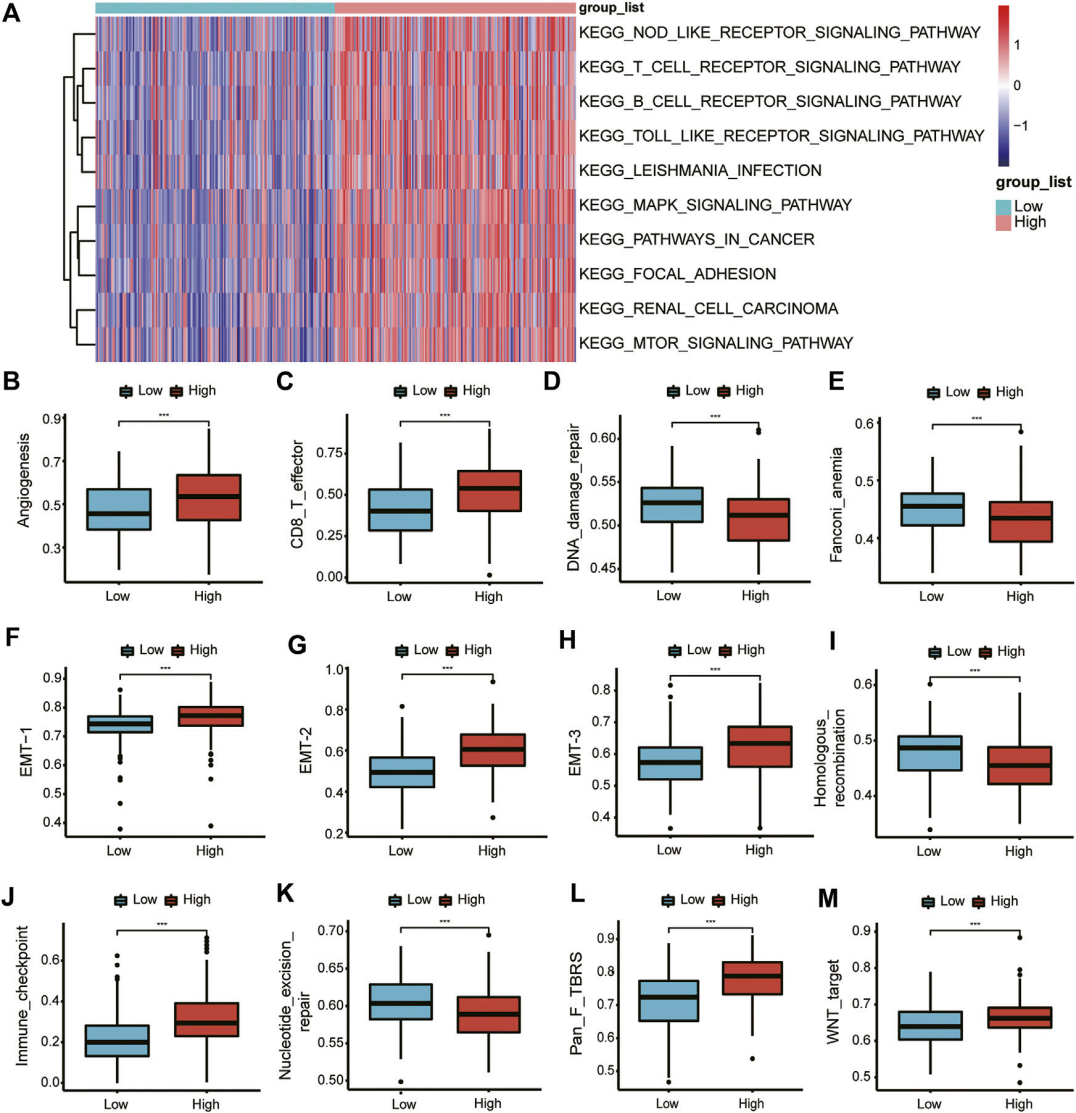
GSVA of DEGs. GSVA of DEGs **(A)**. Differences in various pathways between the high- and low-expression groups **(B–M)**.

To further analyze the difference between the high- and low-expression groups, we selected the 10 pathways with the most significant differences to display as a heat map (Figure 5A). In the high-SLC2A3 expression group, the immune-related pathways, such as the T cell receptor signaling pathway, B cell receptor signaling pathway, and Toll-like receptor signaling pathway, were significantly enriched.

Subsequently, we analyzed the differences in different biological function pathways between the high- and low-expression groups. Angiogenesis, CD86_T cells, EMT-1, EMT-2, and EMT-3, immune checkpoints, fibroblast TGFβ, and Wnt pathways were highly enriched in the high-expression group. In contrast, DNA damage repair, Fanconi anemia, homologous recombination, and nucleotide excision repair were highly enriched in the low-expression group (Figures 5B–M).

### PPI network of SLC2A3 and its interaction with miRNAs

**FIGURE 6:**
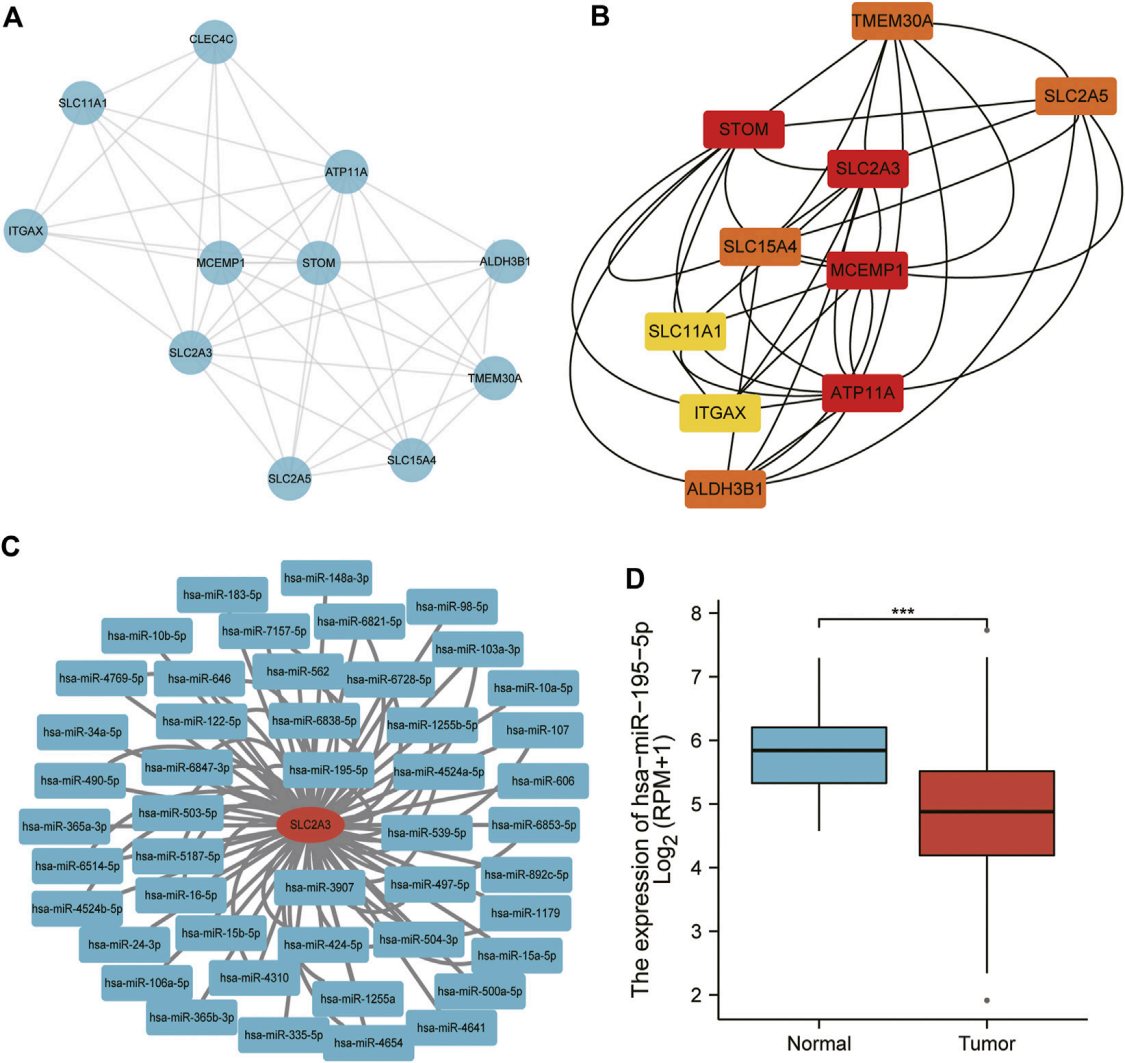
PPI network and miRNA analysis of SLC2A3. The PPI network was visualized using Cytoscape software **(A)** and optimized using the CytoHubba plugin **(B)** Predicted miRNAs interacting with SLC2A3 are shown **(C)**, and miR-195-5p expression was analyzed in gastric cancer **(D)**.

We further analyzed the correlation between SLC2A3 and other molecules. We inputted the SLC2A3 gene into the STRING database and visualized its interactions using Cytoscape software (Figure 6A). After calculating the correlation between molecules using the CytoHubba plugin, we selected the top 10 genes with significant differences to further show their internal relationships (Figure 6B).

The miRNAs interacting with SLC2A3 were predicted using the miRTarBase database, and we obtained 79 miRNAs (Figure 6C), two of which were verified to bind to SLC2A3 *via* luciferase reporter gene experiments. The expression of miR-195-5p was significantly decreased in gastric cancer tissues compared with adjacent normal tissues (*p* < 0.0001; Figure 6D). These findings suggest that there is a close relationship between SLC2A3 and miR-195-5p in gastric cancer.

### Immune cell infiltration analysis

**FIGURE 7:**
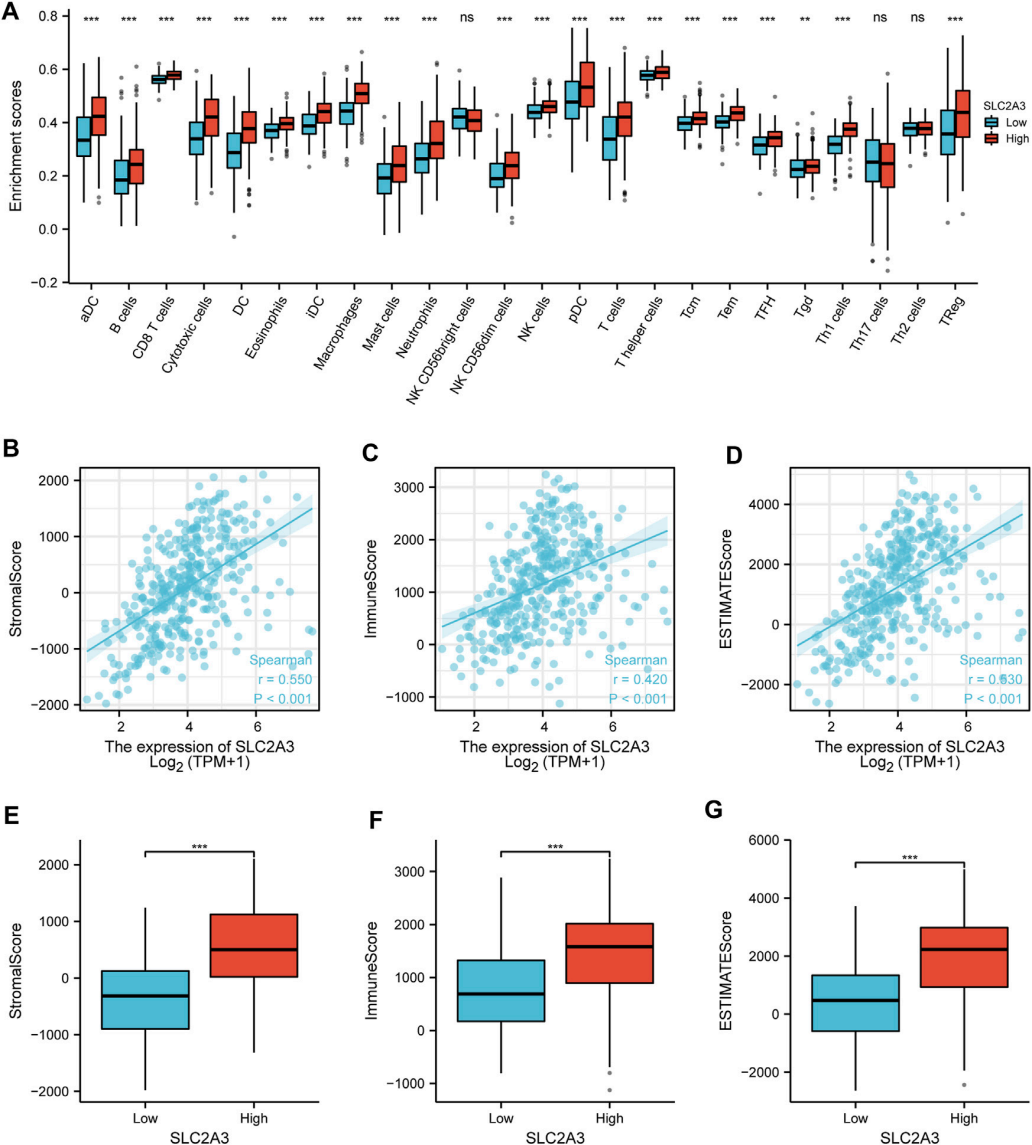
Immune cell infiltration. **(A)** Relationship between the expression of SLC2A3 and immune cell infiltration. Relationship between the expression of SLC2A3 and StromalScore **(B)**, ImmuneScore **(C)**, and ESTIMATEScore **(D)**. Differences in StromalScore **(E)**, ImmuneScore **(F)**, and ESTIMATEScore **(G)** were analyzed between the high- and low-expression groups.

The results of the immune cell infiltration analysis showed that there were significant differences in almost all immune cells (except NK CD56bright, Th17, and Th2 cells) between the high- and low-expression groups (Figure 7A). Most of the immune cells showed significantly higher infiltration in the high-expression group. ESTIMATE analysis results showed that the expression of SLC2A3 was positively correlated with the StromalScore (r = 0.550, *p* < 0.001; Figure 7B), ImmuneScore (r = 0.420, *p* < 0.001; Figure 7C), and ESTIMATEScore (r = 0.530, *p* < 0.001; Figure 7D) calculated using the ESTIMATE algorithm. Further analysis indicated that the StromalScore (*p* < 0.001; Figure 7E), ImmuneScore (*p* < 0.001; Figure 7F), and ESTIMATEScore (*p* < 0.001; Figure 7G) were significantly higher in the high-expression group than in the low-expression group. These results suggest that SLC2A3 is closely associated with immune cell infiltration.

### Construction of a clinical prognosis prediction model

**FIGURE 8:**
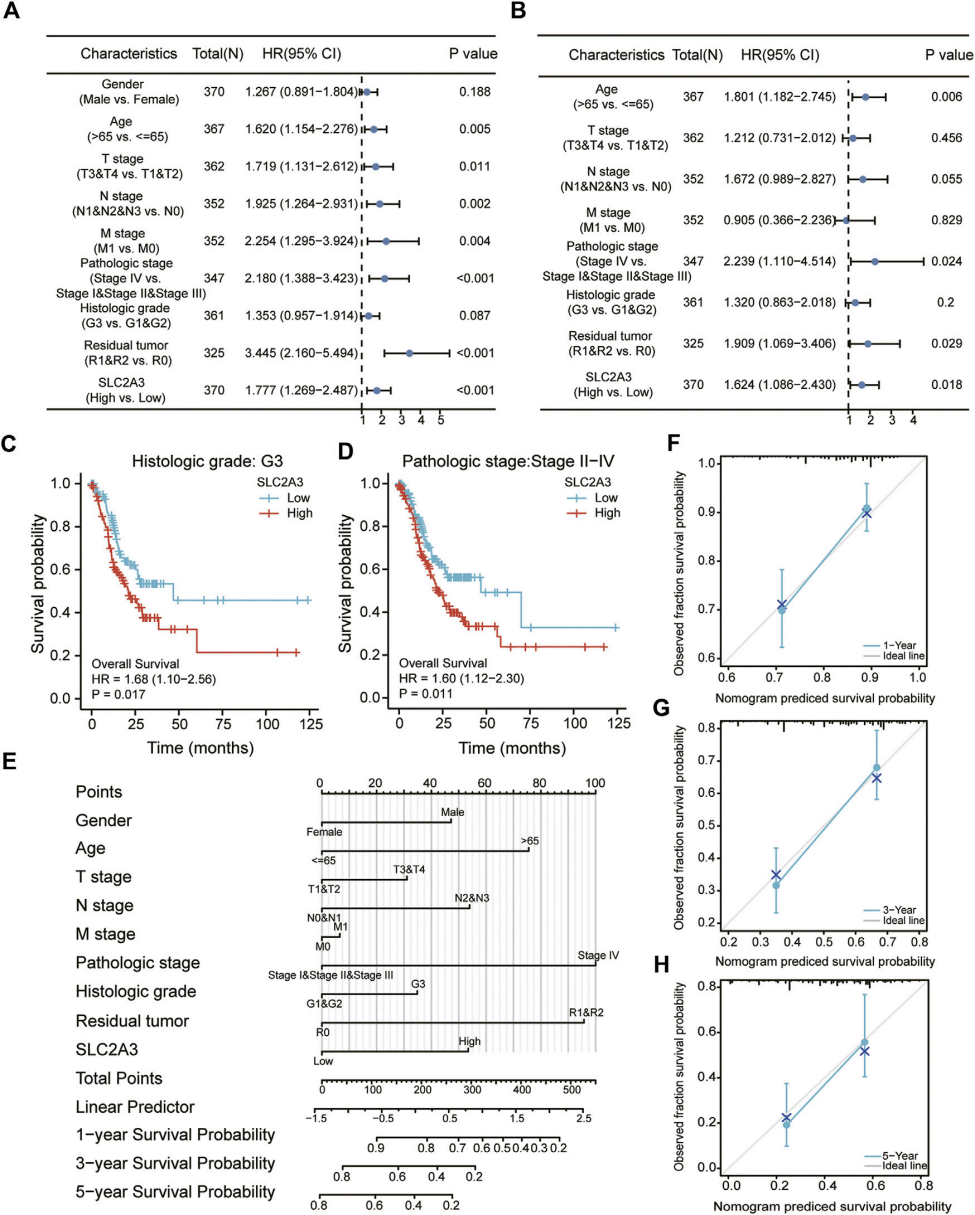
Construction of the clinical prognosis prediction model. Univariate **(A)** and multivariate **(B)** Cox regression analyses. Survival analyses between high- and low-expression groups in the G3 **(C)** and stage II–IV **(D)** groups. Nomogram **(E)** and calibration curve at 1, 3, and 5 years **(F–H)** for prognostic prediction.

Univariate and multivariate Cox regression analyses were performed to explore the relationship between SLC2A3 expression and clinicopathological parameters. In the univariate Cox regression analysis, SLC2A3 was found to be a poor prognostic factor for gastric cancer (hazard ratio: 1.777, 95% confidence interval: 1.269–2.487, *p* < 0.001; Figure 8A), as well as age, TNM stage, pathologic stage, and residual tumor. Similarly, in the multivariate Cox regression analysis, SLC2A3 was a poor prognostic factor for gastric cancer (hazard ratio: 1.624, 95% confidence interval: 1.086–2.430, *p* = 0.018; Figure 8B), as well as age, pathologic stage, and residual tumor. Subsequently, in the subgroup survival analysis, we found that patients in the high-expression group had a poorer prognosis in G3 grade (*p* = 0.017; Figure 8C). Similarly, the high expression level of SLC2A3 was also a poor prognostic factor in the II–IV subgroup of tumor pathological staging (*p* = 0.011; Figure 8D).

We then constructed a clinical prognostic model based on the results obtained from Cox regression analysis, and the results indicated that the higher the expression value of SLC2A3, the lower the probability of survival for patients at 1, 3, and 5 years. Meanwhile, female sex, younger age, lower TNM stage, lower pathologic stage, lower histologic grade, and smaller residual tumor (Figure 8E) were associated with higher survival probability for patients. To evaluate the predictive ability of the model, calibration curves were drawn to show the prediction results at 1, 3, and 5 years. The abscissa is the survival probability predicted by the model, the ordinate is the observed survival probability, and the gray diagonal line is the ideal situation line. The blue lines and dots represent the model predictions at different time points. The calibration charts showed that the model had good predictive ability at 1, 3, and 5 years (Figures 8F–H).

## Discussion

Gastric cancer is one of the most common gastrointestinal tumors and has high morbidity and mortality, threatening human life and health (Machlowska et al., 2020). With the continuous improvement of medical diagnosis and treatment technology, postoperative adjuvant chemotherapy can significantly improve the survival time of patients with advanced gastric cancer (Sexton et al., 2020). However, postoperative recurrence and metastasis problems are still important factors affecting the prognosis of gastric cancer, and the 5-year survival rate is only 20%–35% (Johnston and Beckman, 2019). In recent years, the screening basis of predictive biomarkers for gastric cancer treatment has entered the promoter methylation level (Chen et al., 2021). Therefore, identifying key biomarkers and targets affecting prognosis remains a significant research focus, which will contribute to the clinical outcomes of patients with gastric cancer.

SLC2A3, a ferroptosis marker (Jiang et al., 2017), was found to be highly expressed in gastric cancer, colon cancer, and other tumors. Its overexpression was associated with poor survival and was an unfavorable prognostic indicator for patients with gastric cancer, which was consistent with previous research studies (Chen et al., 2018; Yao et al., 2020). A study on colorectal carcinoma also underscored that upregulation of the SLC2A3 gene was associated with decreased overall and disease-free survival in patients with colorectal cancer, suggesting that determination of SLC2A3 expression might be useful for predicting the prognosis of these patients (Kim et al., 2019). This further demonstrates the important role of SLC2A3 in predicting tumor prognosis; however, the precise mechanism remains elusive.

A previous study in colorectal cancer showed that SLC2A3 could regulate the epithelial–mesenchymal transition (EMT) classical pathway and PD-L1-mediated immune responses (Gao et al., 2021), which was consistent with our research results. Unlike the findings of this study, the results of our functional enrichment analysis showed that SLC2A3 might be related to cytokine–cytokine receptor interaction, EMT, T cell receptor signaling pathway, B cell receptor signaling pathway, and immune checkpoints. Likewise, these pathways had a significant difference between the high-expression group and the low-expression group, as was well-known that EMT was one of the important reasons for tumor metastasis and poor prognosis (Lin et al., 2020). Therefore, based on these results, we inferred that a high SLC2A3 expression level leads to an unfavorable prognosis in gastric cancer, which might be associated with EMT.

Based on the results of the immune microenvironment analysis, we also found that multiple immune cells were significantly different between the high- and low-SLC2A3 expression groups and that StromalScore, ImmuneScore, and ESTIMATEScore were closely related to SLC2A3 expression. These results suggest that SLC2A3 is involved in immunological regulation. This is consistent with the results of a previous study; the analysis with global gene expression profiling of human colorectal cancer cell lines showed that the expression of SLC2A3 was positively correlated with CD4^+^ and CD8^+^ T cells (Gao et al., 2021). In addition, transfection of SW480 and RKO cells with SLC2A3 siRNA significantly attenuated PD-L1 expression. These results strongly suggest that SLC2A3 may be involved in the immune response and immune checkpoint regulation of multiple cancers.

In addition, analysis of SLC2A3-related miRNAs showed that SLC2A3 is closely related to multiple miRNAs, such as miR-195-5p, miR-106a-5p, miR-424-5p, and miR-16-5p. The correlation of miR-106a-5p and SLC2A3 has been demonstrated in human glioma, and inhibition of SLC2A3 by miR-106a attenuated cell proliferation, inhibited glucose uptake, and conferred a favorable survival for patients with glomerular basement membrane disease (Dai et al., 2013). This is also in line with our abovementioned finding that high SLC2A3 expression leads to poor prognosis in patients with gastric cancer. Moreover, a study of human bladder cancer T24 cells reported that miR-195-5p directly targeted the 3′-untranslated region of GLUT3 and downregulated GLUT3 (protein encoded by SLC2A3) expression to decrease glucose uptake, inhibit cell growth, and promote cell apoptosis (Fei et al., 2012). These findings indicate that SLC2A3 is regulated by a variety of miRNAs, particularly miR-195-5p, miR-106a-5p, miR-424-5p, and miR-16-5p, which affect its function. In contrast, our research findings in gastric cancer suggested that there was a close relationship between SLC2A3 and miR-195-5p, indicating that the interaction of SLC2A3 and miR-195-5p might occur not only in bladder cancer but also in stomach cancer.

## Conclusion

Taken together, our findings show that SLC2A3, a ferroptosis marker, is a prognostic marker for poor outcomes and is associated with multiple immune cells. SLC2A3 is also regulated by multiple miRNAs, thereby affecting its ferroptosis- and transmembrane glucose transport-related functions. Understanding the roles of SLC2A3 and the relationship between ferroptosis and tumor immunity can provide valuable insights for treating patients with gastric cancer.

## Data Availability

Publicly available datasets were analyzed in this study. These data can be found here: 1.https://portal.gdc.cancer.gov 2.https://www.ncbi.nlm.nih.gov/geo/ 3.https://gtexportal.org/home/.
